# Are psychotic-like experiences associated with aberrant prosocial decision-making behavior?

**DOI:** 10.3389/fpsyg.2024.1387678

**Published:** 2024-08-02

**Authors:** Julia Elmers, Tana Gabbert, Bastian David, Jakob Scheunemann, Steffen Moritz

**Affiliations:** ^1^Department of Psychiatry and Psychotherapy, University Medical Center Hamburg-Eppendorf, Hamburg, Germany; ^2^Cognitive Neurophysiology, Department of Child and Adolescent Psychiatry, Faculty of Medicine of the TU Dresden, Dresden, Germany; ^3^Department of Epileptology, University Hospital Bonn, Bonn, Germany

**Keywords:** psychosis, continuum approach, social cognition, schizotypy, altruistic punishment

## Abstract

**Introduction:**

Deficits in social functioning and decision-making are well-documented in schizophrenia, but their relationship with positive symptoms and social conflicts is poorly understood. We created a new paradigm based on the Dictator Game (DG) to explore differences in social decision-making between individuals experiencing high levels of psychotic-like experiences (PLEs), particularly hallucinations and delusions, and controls with less PLEs.

**Methods:**

A large community sample (*N* = 1,161) completed a DG in an online study whereby extreme groups were built based on the *positive* subscale of the CAPE.

**Results:**

Overall, participants experiencing PLEs did not act less prosocial than controls but showed a somewhat aberrant decision-making behavior, particularly a pattern of behaving more prosocial in fair situations and generally favoring punishment over compensation relative to controls. Mediation analyses suggest that measures of empathy and Machiavellism have predictive power for prosocial behavior beyond group status.

**Discussion:**

The present study raises the possibility that individuals with high levels of PLEs may be less able to adapt their behavior to the situation at hand than controls. These irregularities might be due to deficits in social cognition which may elicit conflict, thus compromising social functioning and possibly contributing to the formation of positive symptoms.

## 1 Introduction

Schizophrenia is a heterogeneous disorder with an onset in early adulthood and a largely unknown etiology. It is associated with increased depression and suicide rates (Huang et al., 2018; Sher and Kahn, 2019; McGinty and Upthegrove, 2020) and with sequelae such as (self-) stigmatization (Alonso et al., 2019) and poor functional outcome (Green et al., 2019; Halverson et al., 2019).

A scientific approach to understand the etiology of schizophrenia is the research on prodromal and schizotypy phenomena (Ettinger et al., 2014). According to Lenzenweger (2018), “the term schizotypy refers to a latent personality organization that putatively harbors the liability for schizophrenia and can give rise to a variety of schizophrenia-related phenotypic outcomes” (p. 25). Therefore, schizotypy is similar to schizophrenia in a phenomenological sense, so that schizotypal individuals show an attenuated type of schizophrenia phenomena, like sensory irritation, suspiciousness, and odd behavior not fulfilling the magnitude seen in individuals with diagnosed schizophrenia. These phenomena are now more commonly referred to as psychotic-like experiences (PLEs) and occur much more often among the general population than schizophrenia itself (McGrath et al., 2015).

To this date, it is still unclear whether and how schizophrenia (or PLEs, respectively) and altruism are related. Some studies found positive associations between schizophrenia or paranoia, respectively, and altruistic behavior (Agnati et al., 2012; Horat et al., 2018), however, there are several studies reporting negative correlations (Hanssen et al., 2017; Purushothaman et al., 2019; Raihani et al., 2021) or null results (Wischniewski and Brüne, 2011; McGuire et al., 2017; Claassen et al., 2020).

Since prosocial and altruistic behaviors are crucial for cohabitation in society (Fehr and Fischbacher, 2003), the present study aims to investigate whether cognitive and affective alterations in individuals experiencing PLEs affect prosocial and/or altruistic decision-making behavior in the context of social norm violations in a Dictator Game (DG; e.g., Leder and Schütz, 2018). It is important to mention that *altruism* and *prosocial behavior* are two distinct constructs often misused as synonyms. Prosocial behavior is much more of a superordinate construct for a wide spectrum of actions ought to benefit others and not oneself (Batson, 2012), which may include helping, comforting, sharing, cooperation, and community service. In economic games like the DG, the most frequently studied parameters are altruistic compensation (i.e., give points to an unfairly treated player) and altruistic punishment (i.e., take points from a player, who treated others unfairly). While altruistic compensation is assumed to be based on empathy and altruistically motivated (Leliveld et al., 2012; Hu et al., 2015; Liu et al., 2017; Rodrigues et al., 2018), altruistic punishment is assumed to be mostly strategically motivated to ensure social norms (e.g., fairness) and suggested to be based on anger, feelings of revenge, and striving for dominance (Fehr and Gächter, 2002; Jordan et al., 2016; Rodrigues et al., 2018; Windmann and Hein, 2018). Importantly, punishment in the DG is solely financial and does not imply that a person is prone to aggression or hostility in his or her daily life. Previous research revealed neural (Sugranyes et al., 2011; Wible, 2012; Preckel et al., 2018), cognitive (Waytz et al., 2012; Savla et al., 2013; Majdandžić et al., 2016; Davies and Greenwood, 2018; Green et al., 2019; Halverson et al., 2019), and affective (Bora et al., 2008; Klimecki et al., 2016; Bonfils et al., 2017) alterations in individuals with schizophrenia, as well as their adverse effects on social functioning. Given that neurocognitive research on individuals with schizophrenia has several disadvantages (Moritz et al., 2021) and schizotypy represents an attenuated form of manifest schizophrenia symptoms, this study focused on the latter group with the assumption that individuals experiencing high levels of PLEs will act less prosocial in a modified DG due to their phenomenological similarities to people with schizophrenia. We also assume that these participants will use more costly punishment than altruistic compensation compared to controls possibly arising from higher depression scores like former studies suggest (Wischniewski and Brüne, 2011; Upthegrove et al., 2017). In an exploratory fashion, we will investigate whether the perception of power imbalance between two individuals influences prosocial behavior.

## 2 Methods

### 2.1 Recruiting

We created an online survey in Qualtrics (Provo, Utah, United States) and recruited participants via Amazon Mechanical Turk (MTurk), an online crowdsourcing service where individuals can participate in web-based “human intelligence tasks” (HITs) for money. Data was collected on four separate days in October and November 2020. Advantages of collecting data via MTurk are its cost-effectiveness and the fast recruitment of a high number of study participants with diverse ethnical and socio-economic backgrounds (Buhrmester et al., 2011; Shapiro et al., 2013; Boas et al., 2020). The quality of collected data by MTurk has been confirmed several times (Crump et al., 2013; Thomas and Clifford, 2017; McCredie and Morey, 2019).

Following recommendations by Kees et al. (2017), the primary inclusion criteria for the participants were a minimum age of 18, an US IP address (i.e., only USA residents), and an acceptance rate of 95% for previous tasks. In addition, participants had to pass several validity checks (e.g., processing time or inattentiveness/manipulation checks) before they were included in the analysis set in order to ensure high quality (Aruguete et al., 2019).

### 2.2 Participants

Of 1,271 people who began the survey, 48 did not finish, 39 were excluded because they sped through the survey (defined by a response time of 50% of the median completion time [cutoff: 8.22 min]), and 23 of the remaining participants failed the validity checks (i.e., rating a minimum of two items on the Psychosis Lie Scale with “*totally agree*” and/or a total score above 15 on the Infrequency Scale, see below). All exclusions were made blind to results. After removing a total of 110 (8.6%) participants, 1,161 participants (57.5% females) were included in the analyses and divided into two groups according to the frequency of reported PLEs in the subscale *positive symptoms* of the Community Assessment of Psychotic Experiences (Stefanis et al., 2002). Following Moritz et al. (2017, 2019) participants scoring at least 2 *SD* above the mean (*M* = 26.62, *SD* = 7.09) were assigned to the PLEs-high group (*n* = 66), whereas participants scoring at most 0.5 *SD* above the mean were assigned to the PLEs-low group (*n* = 902) for extreme group comparison. Extreme group design is an often-used method when investigating the latent construct of schizotypy by the number of PLEs (see also Lenzenweger and Korfine, 1994; Hahn et al., 2021). Therefore, the main analyses were conducted with a total sample of 968 participants. All participants provided their written informed consent after receiving a detailed description of the study. The Ethics and Safety Committee of the University Medical Center Hamburg-Eppendorf (Germany) approved the study protocol (LPEK-0206) on October 22, 2020. Participants received 1.50 USD for their participation, independent from their or others behavioral choices in the presented experiment.

### 2.3 Psychometric measures and quality checks

The Community Assessment of Psychotic Experiences (CAPE) was administered to measure the frequency of lifetime PLEs, the subscale *Altruism* of the NEO Personality Inventory-Revised (NEO-PI-R; Costa and McCrae, 2008) to measure altruism as a personality trait, the International positive and negative affect schedule short-form (PANAS-SF; Thompson, 2007) to evaluate the frequency of positive and negative affectivity, and the Toronto Empathy Questionnaire (Spreng et al., 2009) to assess empathy. Additionally, the Trimmed MACH* (Rauthmann, 2013) was used to measure Machiavellian tendencies.

Further, we implemented four items of the Psychosis Lie Scale (Moritz et al., 2013) and six items of an Infrequency Scale (Jackson, 1974) to assess biases due to positive self-presentation and inattentiveness for validity checks.

### 2.4 Procedure

**Figure 1:**
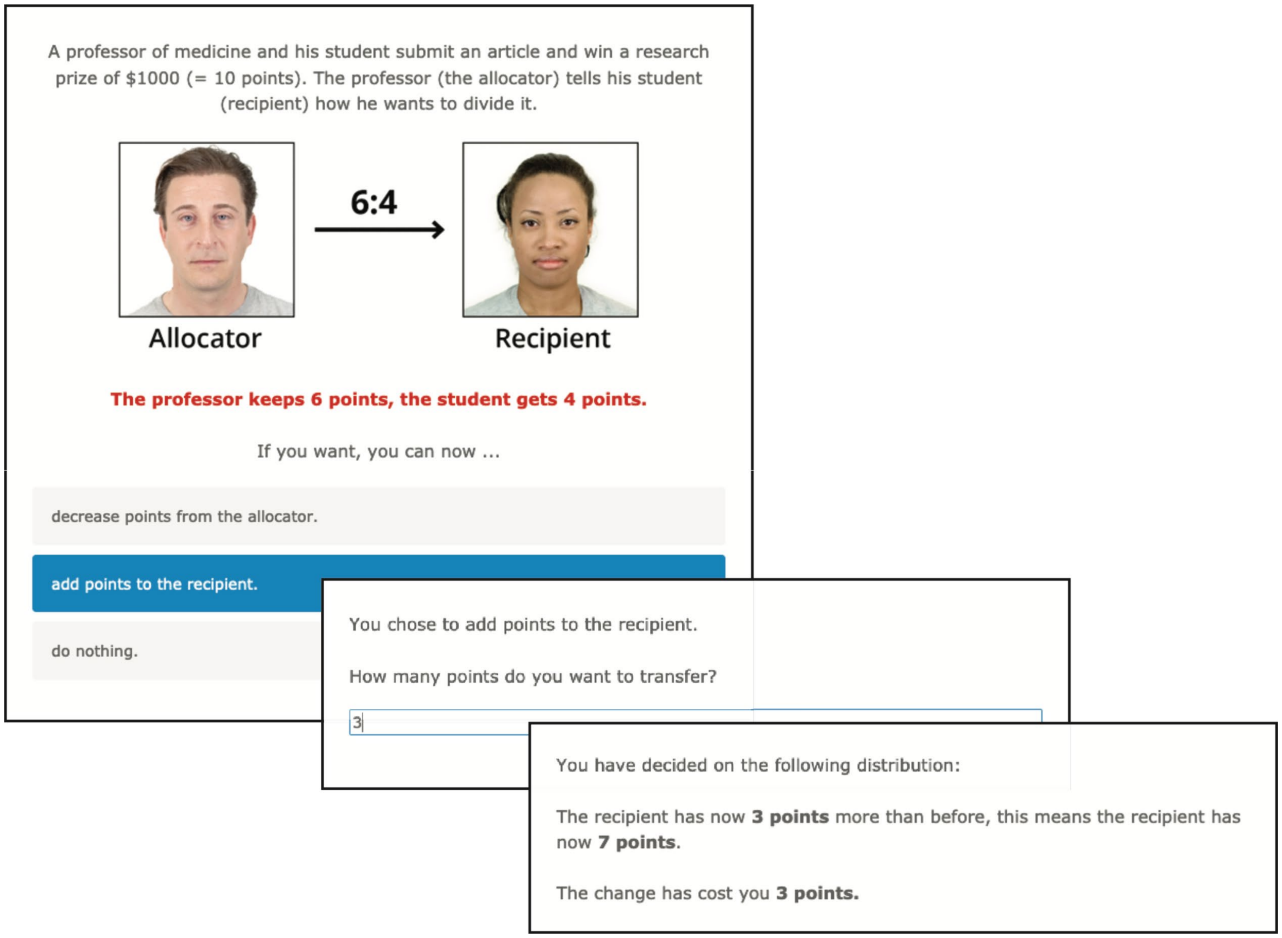
Timeline of screens within an exemplary trial of the Third-Party DG. In this exemplary run, the participant first saw an unfair trial (distribution 6:4) with authority (allocator is a medicine professor) and chose “add points to the recipient” (i.e., compensation). After this, he/she decided on the next page to compensate the recipient with 3 points. The last screen only summarizes the final distribution of points in this trial. Pictures of fictional allocators and receivers were used from the Chicago Face Database (Version 2.0.3; Ma et al., 2015).

We used a modified Third-Party DG with participants as observers to assess altruistic behavior. In our online survey, participants should imagine two fictive scenarios in which two people divide 1,000 US dollars between each other. We designed the paradigm as a 2 × 3 factorial design with the factors *authority* (allocation by a peer person vs. allocation by a professor) and *fairness* (fair offer vs. unfair offer vs. extremely unfair offer). Regarding the new factor authority, we provided two different scenarios. The scenario without power imbalance (no authority) showed two peer persons dividing the money from a lottery win, whereby in the scenario with power imbalance a medicine professor (the allocator) and his or her student (the recipient) divided the prize money for a research article (see Figure 1). The three fairness conditions were determined as follows: fair offer = 5:5, unfair offer = 6:4, extremely unfair offer = 9:1. The procedure was as follows: participants first saw a screen instructing them to imagine one of the two scenarios (i.e., with or without authority) and could than chose to punish the allocator (i.e., “decrease points from the allocator”), compensate the recipient (i.e., “add points to the recipient”) or keep the points (i.e., “do nothing”). After this, the participant could decide on the next page how many points he or she wants to transfer. The last screen summarizes the final distribution of points in this trial (see Figure 1). Participants were explicitly told that their decisions in the game would not influence their endowment at the end of the experiment (i.e., “Your transfer decisions will NOT affect your payment for this HIT”).

To design the scenarios in a more realistic fashion, we followed the set-up by Wischniewski and Brüne (2011) and presented photographs of 36 individuals in the roles of allocator and recipient to the participants. We used pictures from the Chicago Face Database (Ma et al., 2015) because it provides high-resolution, standardized photographs of 597 male and female targets of varying ethnicity and corresponding norming data from 1,087 raters. Only pictures of individuals with neutral face expression and medium rated attractiveness (around 4 on a 7-point Likert scale) were used as more extreme expressions in these features are assumed to influence cooperative and prosocial behaviors (Farrelly et al., 2007; Mussel et al., 2013; Greiner et al., 2014; Bhogal et al., 2017). All participants underwent a total of 18 one-shot trials (i.e., every allocator–recipient pair was shown only once).

### 2.5 Statistical analysis

Analyses were carried out using SPSS, version 27. For the comparison of descriptive group differences, Chi-square tests were used for the comparison of categorical data, while Welch’s *F*-test was used for the comparison of metric data.

Further, we conducted Pearson correlation analyses to explore relationships between questionnaire measures and (prosocial) decision rates in the DG. For the evaluation of decision-making behavior in the DG, punishment, compensation, and keep rates (i.e., total number of times a participant decided on each of the three options within the 18 trials) were calculated first. Afterwards three-way mixed ANOVAs were used to compare differences in punishment, compensation, and keep rates between groups. Levels of significance for all analyses were *p* = 0.05 (two-tailed).

We also investigated direct and indirect effects of empathy and Machiavellism on (prosocial) decisions in the DG with mediator analyses using Hayes’ process macro in SPSS (Model 6; Hayes, 2017).

To check for socio-demographic influences, all statistical procedures were repeated with an age-, gender-, and education-matched sample (matching was done blind to results) consisting of 128 participants (*n* = 64 in each group). We will refer to this second exploratory sample as *matched sample* in the following. Results of the matched sample are only mentioned in case of significant differences to the main analysis (see 4.5).

Additionally, to assess the internal consistency measurements for all questionnaire scales in both samples, we calculated Cronbach’s *α*.

## 3 Results

### 3.1 Descriptive statistics

The groups did not differ in gender and educational level, but in age, educational duration, race, and monthly income (Table 1). Further, there was a significant difference between the two groups regarding self-reported psychiatric lifetime diagnoses with a higher prevalence in the PLEs-high group than the PLEs-low group (Table 2).

**Table 1:** Demographic characteristics and group differences.

	PLEs-low (*n* = 902)	PLEs-high (*n* = 66)	Group differences	
Gender			*χ*^2^(3) = 6.75	*p* = 0.08
Female	57.6%	42.4%		
Male	41.1%	56.1%		
A different term/I wish not to answer^a^	1.3%	1.5%		
Age	40.9 (*SD* = 12.9) [18–89]	32.7 (*SD* = 8.9) [19–64]	Welch’s *F*(2, 86.41) = 48.49	*p* < 0.001
Monthly Income			*χ*^2^(3) = 29.06	*p* < 0.001
Less than $500	12.4%	24.2%		
$500–1.000	9.4%	25.8%		
$ 1.000 – $2.000	24.7%	19.7%		
Over $2.000	53.4%	30.3%		
Level of education			*χ*^2^(7) = 5.44	*p* = 0.607
Less than high school	0.4%	1.5%		
High school graduate	17.3%	19.7%		
College	21.3%	21.2%		
Bachelor’s Degree	42.2%	43.9%		
Master’s Degree	13.0%	13.6%		
Professional degree	2.5%	0.0%		
Doctorate	2.4%	0.0%		
Other	0.8%	0.0%		
Years of education	14.6 (*SD* = 4.5) [2–30]	12.5 (*SD* = 5.2) [1–22]	Welch’s *F*(2, 69.51) = 10.39	*p* < 0.001
Race/ethnicity^b^			*χ*^2^(8) = 47.02	*p* < 0.001
American Indian or Alaska Native	0.7%	3.0%		
Asian or Asian American	8.5%	10.6%		
Black or African American	7.8%	27.3%		
Latino or Hispanic	5.9%	10.6%		
Native Hawaiian	0.6%	0.0%		
White or European American	77.6%	60.6%		
Neither/I wish not to answer^a^	2.3%	0.0%		

^a^Answers were pooled. ^b^Multiple answers were possible.

**Table 2:** Self-reported lifetime diagnoses.

	PLEs-low (*n* = 902)	PLEs-high (*n* = 66)
No psychiatric disorder	56.5%	21.2%
Schizophrenia	0.3%	7.6%
Depression	25.2%	54.5%
Anxiety Disorder	23.7%	37.9%
Attention deficit hyperactivity disorder	5.5%	15.2%
Posttraumatic stress disorder	5.8%	18.2%
Obsessive-compulsive disorder	2.8%	6.1%
Bipolar Disorder	2.7%	18.2%
Eating Disorder	2.7%	13.6%
Substance Abuse	3.5%	16.7%
Personality Disorder	0.6%	10.6%
Other	4.5%	3.0%

Multiple answers were possible. Group difference *χ*^2^(12) = 271.04, *p* < 0.001.

### 3.2 Questionnaire measurements and prosocial behavior

All questionnaire scales showed a good internal consistency for both samples (Supplementary Table S1). Individuals in the PLEs-high group displayed greater depression, Machiavellism, negative affect, and distrust scores, as well as lower empathy and altruism scores than individuals in the PLEs-low group. Additionally, one-way comparisons of experimental data with Welch’s ANOVA showed differences in prosocial behavior between groups in regard to fairness with elevated prosocial behavior (punishment and compensation rates) with increasing unfairness (Table 3). Results show mainly medium (Cohen’s *d* > 0.5) to high (Cohen’s *d* > 0.8) effect sizes. Confidence intervals (95%) were calculated following (Hemmerich, 2016).

**Table 3:** Means and standard deviations of questionnaire measures and experimental data.

	PLEs-low (*n* = 902)	PLEs-high (*n* = 66)	Group differences	
Psychopathology				
CAPE				
Subscale positive	24.9 (3.1)	49.7 (5.5)	Welch’s *F*(2, 68.12) = 1317.21	*p* < 0.001, *d* = 7.48 (95%–CI[7.063; 7.897])
Subscale depression	14.4 (3.5)	21.2 (4.8)	Welch’s *F*(2, 70.09) = 128.08	*p* < 0.001, *d* = 1.89 (95%–CI[1.626; 2.154])
TEQ	46.8 (8.6)	37.8 (8.9)	Welch’s *F*(2, 74.24) = 64.07	*p* < 0.001, *d* = −1.04 (95%–CI[−1.295; −0.785])
NEO-PI-R Altruism	22.4 (4.0)	20.7 (4.5)	Welch’s *F*(2, 72.91) = 8.41	*p* = 0.005, *d* = −0.42 (95%–CI[−0.671; −0.169])
Trimmed MACH	6.7 (4.1)	11.9 (3.1)	Welch’s *F*(2, 82.48) = 171.24	*p* < 0.001, *d* = 1.29 (95%–CI[1.033; 1.547])
Distrust	5.8 (3.0)	7.1 (2.7)	Welch’s *F*(2, 77.52) = 14.37	*p* < 0.001, *d* = 0.44 (95%–CI[0.189; 0.691])
PANSS				
Subscale negative	8.7 (2.6)	14.6 (4.2)	Welch’s *F*(2, 68.52) = 121.03	*p* < 0.001, *d* = 2.16 (95%–CI[1.892; 2.428])
Subscale positive	16.9 (3.9)	17.2 (3.2)	Welch’s *F*(2, 80.03) = 0.60	*p* = 0.440
Dictator Game^a^				
Fair Trials				
Punishment	0.05 (0.35)	0.41 (0.80)	Welch’s *F*(2, 66.86) = 13.01	*p* = 0.001, *d* = 0.91 (95%–CI[0.656; 1.164])
Compensation	0.15 (0.62)	0.73 (1.42)	Welch’s *F*(2, 66.81) = 10.94	*p* = 0.002, *d* = 0.83 (95%–CI[0.577; 1.083])
Keep	5.80 (0.77)	4.86 (1.88)	Welch’s *F*(2, 66.59) = 16.34	*p* < 0.001, *d* = −1.06 (95%–CI[−1.315; −0.805])
Unfair Trials				
Punishment	0.88 (1.49)	1.35 (1.66)	F(1, 966) = 5.90	*p* = 0.015, *d* = 0.31 (95%–CI[0.059; 0.561])
Compensation	2.87 (2.29)	2.27 (1.99)	Welch’s *F*(2, 78.03) = 5.46	*p* = 0.022, *d* = −0.26 (95%–CI[−0.511; −0.009])
Keep	2.24 (2.21)	2.38 (2.33)	F(1, 966) = 0.23	*p* = 0.631
Extremely Unfair Trials				
Punishment	1.77 (2.12)	2.06 (2.09)	F(1, 966) = 1.19	*p* = 0.277
Compensation	3.50 (2.33)	2.53 (2.12)	Welch’s *F*(2, 76.96) = 12.70	*p* = 0.001, *d* = −0.42 (95%–CI[−0.671; −0.169])
Keep	0.73 (1.59)	1.42 (1.96)	Welch’s *F*(2, 71.36) = 7.52	*p* = 0.008, *d* = 0.43 (95%–CI[0.179; 0.681])

If assumption of homoscedasticity (Levene statistic) was violated, Welch’s ANOVA was conducted. ^a^A higher mean value indicates that this type of decision was made more often for an overall of six trials for each fairness condition. CAPE = Community Assessment of Psychotic Experiences; TEQ = Toronto Empathy Questionnaire; NEO-PI-R = The Revised NEO Personality Inventory; PANSS = Positive and Negative Syndrome Scale.

We conducted a correlational analysis for questionnaires and experimental measurements across the main sample (*N* = 968; Supplementary Table S2). The analysis revealed significant relationships (*ps* < 0.01, 2-sided) for schizotypy with depression (*r* = 0.51), distrust against authorities (*r* = 0.19), Machiavellism (*r* = 0.39), negative affect (*r* = 0.53), altruism (*r* = −0.13), and empathy (*r* = −0.26). We found significant correlations between schizotypy and punishment, compensation, and keep rates. An unexpected pattern of results for prosocial behavior emerged: In fair trials, increased proneness to PLEs was associated with a *higher* frequency of punishment (*r* = 0.24) and compensating behavior (*r* = 0.17).

Correlations between other questionnaire measures and experimental data showed weak positive correlations of empathy and compensating behavior in unfair trials (*r* = 0.12) and in extreme unfair trials (*r* = 0.16). Machiavellism was negatively correlated with the frequency of compensation in unfair trials and (*r* = −0.14) and in extreme unfair trials (*r* = −0.15) and partly positively correlated with punishment.

### 3.3 Frequency of decision-making behaviors between groups

For each decision in the DG (i.e., compensation, punishment, and keep), we calculated a separate 2 × 2 × 3 mixed ANOVA, resulting in three different ANOVAs. In each ANOVA, the experimental conditions fairness (3 levels) and authority (2 levels) served as within-subject factors, whereas group status (2 levels) served as the between-subject factor. To correct for violations of sphericity, Greenhouse–Geisser adjustment was used.

For compensation, the analysis revealed descriptively greater compensation behavior in the PLEs-low group (*p* = 0.057, *η*_p_^2^ = 0.004), however, this was not statistically significant. As expected, there was a significant main effect of fairness, *F*(1.99, 1924.87) = 170.99, *p* < 0.001, *η*_p_^2^ = 0.150 (95%–CI[0.122;0.178]) with higher compensation rates as unfairness increased. There was no main effect for authority (*p* = 0.61). Analysis revealed an interaction effect between fairness and group with stronger rising compensation rates and greater descriptive differentiation between fair and unfair trials in the PLEs-low group, *F*(1.99, 1924.87) = 20.31, *p* < 0.001, *η*_p_^2^ = 0.015 (95%–CI[0.009;0.034]). There was also an interaction effect between fairness and authority, *F*(1.75, 1692.35) = 7.08, *p* = 0.001, *η*_p_^2^ = 0.007 (95%–CI[0.001;0.017]), with higher compensation rates in fair and extreme unfair trials with authority but lower compensation rates in unfair trials with authority.

For punishing behavior there was a significant main effect of group with slightly more punishment behavior observed in the PLEs-high than in the PLEs-low group, *F*(1, 966) = 7.55, *p* = 0.006, *η*_p_^2^ = 0.008 (95%–CI[0.001;0.022]). Additionally, there were two significant main effects of within-subject factors; higher punishment rates occurred with increased levels of unfairness, *F*(1.73, 1672.30) = 100.08, *p* < 0.001, *η*_p_^2^ = 0.094 (95%–CI[0.069;0.120]) and in trials without authority, *F*(1, 966) = 9.00, *p* = 0.003, *η*_p_^2^ = 0.009 (95%–CI[0.001;0.025]). An interaction effect for authority and group revealed that punishment rates differed significantly in trials with and without authority in the PLEs-low group but not in the PLEs-high group, *F*(1, 966) = 4.18, *p* = 0.041, *η*_p_^2^ = 0.004 (95%–CI[0.000; 0.016]).

There was no main effect of group for keep behavior (*p* = 0.556) but in line with other results, keep rates decreased with elevated levels of unfairness, *F*(1.79, 1730.34) = 616.05, *p* < 0.001, *η*_p_^2^ = 0.389 (95%–CI[0.356;0.420]). Further, higher keep rates were found in trials with authority, *F*(1, 966) = 26.78, *p* < 0.001, *η*_p_^2^ = 0.027 (95%–CI[0.010;0.050]). An interaction effect between fairness and authority showed that keep decisions in unfair and extreme unfair trials had a wider distribution of mean keep rates between different authority conditions than in fair trials, *F*(1.53, 1474.55) = 13.78, *p* < 0.001, *η*_p_^2^ = 0.014 (95%–CI[0.004;0.028]). Additionally, there were interactions effects between group status and levels of fairness {*F*(1.79, 1730.34) = 18.02, *p* < 0.001, *η*_p_^2^ = 0.018 (95%–CI[0.008;0.032])} and authority {*F*(1, 966) = 4.37, *p* = 0.037, *η*_p_^2^ = 0.005 (95%–CI[0.000;0.017])}. Thereby, variance of mean values in keep rates in different conditions were descriptively greater in the PLEs-low group compared to the PLEs-high group.

**Figure 2:**
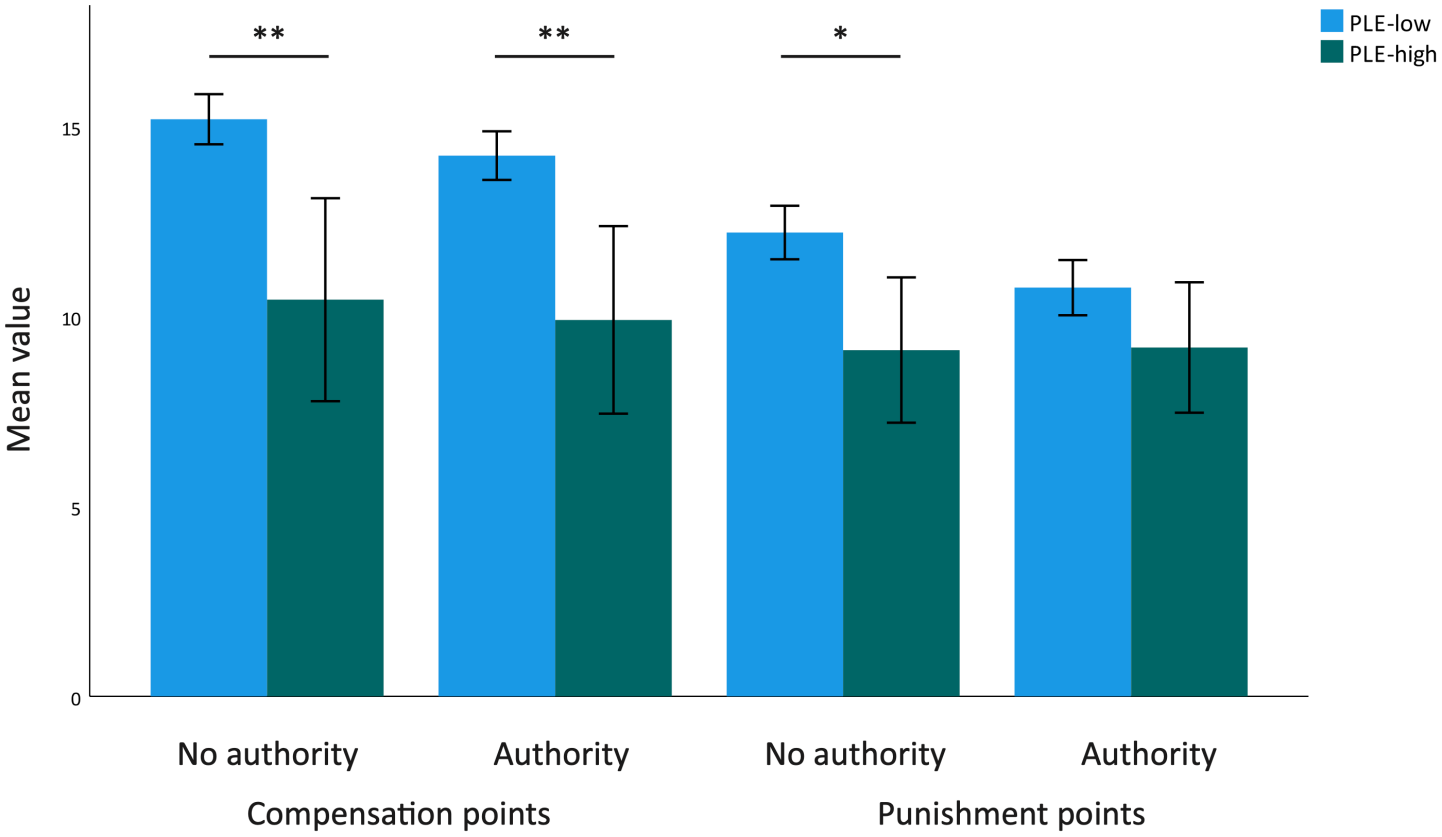
Mean number of compensation and punishment points in extremely unfair trials (*N* = 968). Error bars indicate 95% confidence interval. *p < 0.01, **p = 0.001.

An exploratory examination of the number of transferred punishment and compensation points (i.e., the strength of punishment or compensation) revealed significant differences only in extremely unfair trials (Figure 2). The PLEs-low group transferred more compensation points in trials with authority, Welch’s *F*(2, 48.05) = 11.45, *p* = 0.001 and without authority, Welch’s *F*(2, 45.21) = 11.86, *p* = 0.001 compared to the PLEs-high group. Additionally, individuals in the PLEs-low group also transferred significantly more punishment points in trials without authority, Welch’s *F*(2, 44.11) = 9.16, *p* = 0.004.

### 3.4 Mediation of empathy and Machiavellism on decision-making behavior

As previous literature suggests an immense influence of empathy on altruistic and prosocial behavior (Batson et al., 1989; Leliveld et al., 2012; Hu et al., 2015; Klimecki et al., 2016; Liu et al., 2017), mediation analyses were carried out to investigate if the association between schizotypy (predictor) and decision behavior (outcome) was (at least partially) explained by empathy (mediator 1). Further, because of its significant correlations with punishment and compensation outcomes, Machiavellism (mediator 2) was also included as a mediator for explanatory evaluation.

**Figure 3:**
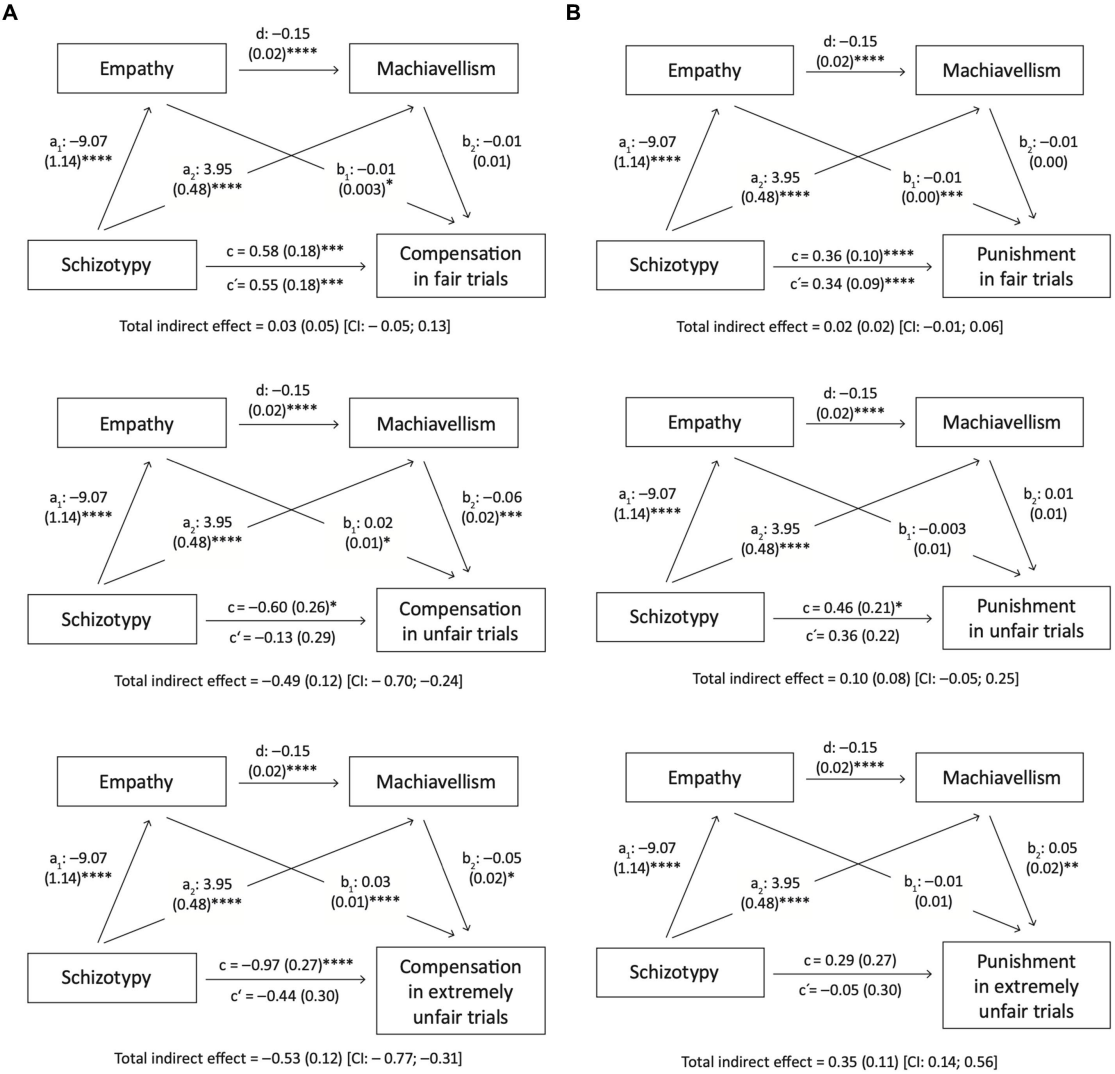
Mediation analyses for **(A)** compensation and **(B)** punishment rates in fair, unfair and extremely unfair trials (*N* = 968). Models of mediation analyses with schizotypy as predictor, empathy and Machiavellism as mediators, and compensation/punishment rates in different fairness conditions as criterions. Beta-coefficients and standard errors for direct effects are displayed next to regression pathways. c’ = direct effect, c = total effect. *p < 0.05, **p < 0.01, ***p < 0.005, ****p < 0.001.

We found mediation effects (i.e., total indirect effect did not include zero) for compensation in unfair and extremely unfair trials with decreased compensation rates in the PLEs-high group mediated through lower empathy and higher Machiavellism scores (Figure 3A). A mediation effect for punishment was only found in extremely unfair trials with higher punishment scores in the PLEs-high group mediated through higher Machiavellism scores (Figure 3B).

### 3.5 Results of analysis in the matched sample

To account for the effects of age, gender, and education, we repeated the statistical analyses with a matched sample (*n* = 128). Results of correlational analysis and most of the results regarding decision-making behavior remained largely unchanged. Except for the interaction effect of fairness and authority (*p* = 0.105), all other effects remained significant for compensation rates, with even somewhat larger effect sizes. For punishment rates, the main effect of group and the interaction effect no longer reached significance. Effects for keep behavior remained significant (except for the interaction between authority and group) with increased effect sizes.

Repeated mediation analyses within the matched sample revealed only stable effects for compensation and punishment rates in fair trials. Mediation effects in other trials no longer reached significance (Supplementary Figure S3).

## 4 Discussion

This study aimed to investigate whether individuals experiencing PLEs (often labeled as *highly schizotypal* in research) act less prosocial and altruistic than controls in a modified version of the DG. In addition, psychological constructs such as empathy, negative affect, and Machiavellism were measured in self-report questionnaires and used in subsequent mediation analyses.

Results from correlational analyses revealed a significant relationship between PLEs and measures of empathy, negative affect, and depression corroborating prior research in schizophrenia (Bora et al., 2008; Baez et al., 2013; Mote and Kring, 2019), as well as an association between PLEs with Machiavellism. Correlational measures were also in line with previous findings concluding that compensating behavior (i.e., helping the victim) is driven by empathy (Leliveld et al., 2012; Hu et al., 2015; Böckler et al., 2016; Liu et al., 2017; Rodrigues et al., 2018). Moreover, the present study showed a positive correlation between Machiavellism and punishment, but a negative correlation with compensation. This supports the idea that punishment is linked with the desire to demonstrate power and supports the idea that punishment may be based on feelings of anger and strategic action (Fehr and Gächter, 2002; Böckler et al., 2016; Jordan et al., 2016; Rodrigues et al., 2018; Windmann and Hein, 2018).

To our knowledge, this is the first study investigating the relationship between PLEs and prosocial behavior under consideration of varying levels of fairness and authority in a Third-Party DG. Only two studies investigated altruistic punishing behavior in schizophrenia with a Third-Party DG in which participants acted as observers (Wischniewski and Brüne, 2011; Claassen et al., 2020). Although these studies did not find a significant difference in punishing behavior between individuals with schizophrenia and healthy controls, the current study revealed an aberrant pattern of prosocial decision-making behavior in PLEs-high individuals experiencing high levels of PLEs under certain circumstances. This is particularly interesting because psychotic phenomena are usually less pronounced in schizotypal individuals than in people with schizophrenia, and yet our study was able to show an aberrant behavior in subclinical manifestations.

As expected, prosocial behavior (i.e., levels of compensation and punishment) increased with higher levels of unfairness in both groups. Contrary to findings from Liu et al. (2018), participants acted less prosocial (i.e., they made more keep decisions) in trials with authority. These contradictory results could arise from several factors. While Liu et al. (2018) used rival student associations to divide participants into an in-group versus an out-group, the current study design used a scenario with no direct reference to reality and a greater difference in socio-economic status (i.e., higher status of medicine professors than students). Therefore, factors such as respect or admiration for specific leaders may have influenced decisions in our study. However, under certain circumstances, groups differed in their prosocial behavior in trials with and without authority in the current study. Positive correlations of self-reported distrust against authority and PLEs, together with no significant differentiation in punishment behavior between trials with and without authority in the PLEs-high group, may suggest that individuals experiencing high levels of PLEs do not hesitate to punish norm-violating authorities (regardless of the level of fairness) because they feel supported in their distrust against them (see also Fett et al., 2012). Interestingly, when looking more closely at sub-analyses, a main effect of authority was only found for punishment and not for compensation. Therefore, prosocial behaviors based on empathy may not depend on environmental factors to the same extent as punishment behaviors.

Overall, analyses of variance revealed an interesting pattern of decision-making behavior in the PLEs-high group. First, the main hypothesis could not be fully confirmed as groups did not differ in their overall rate of prosocial behavior (i.e., no differences in keep decisions between groups). In line with our assumptions, the PLEs-high group favored punishment over compensation. This preference was not due to increased depression scores as suggested by Wischniewski and Brüne (2011), but rather due to increased Machiavellianism and decreased empathy scores in the PLEs-high group. Second, participants experiencing PLEs showed more prosocial behavior in fair trials than controls. This was somewhat surprising because we assumed that decision-making behavior would not differ in fair trials. Nevertheless, abnormal decision-making behavior in the sense of excessive prosocial behavior in fair situations has been reported for individuals with schizophrenia before, leading to the assumption that patients think less strategically than healthy controls (Robson et al., 2020). Third, an overarching pattern emerged in which decisions made by individuals with high levels of PLEs depended less on context than decisions made by controls. This behavior pattern may result from biased or impaired social cognition reported earlier in individuals prone to psychosis (Cowan et al., 2019).

Findings from subsequent mediation analysis were in line with the aforementioned results. Current findings thus emphasize the idea that empathy and Machiavellism may have more information value than the diagnostic status itself like it was shown for mentalizing before (Claassen et al., 2020). Given that mediation effects did not reach significance in the matched sample, this idea and the possible impact of other variables (e.g., gender or education) need further investigation.

### 4.1 Limitations

The advantage of the current study design lies in the concurrent consideration of various variables with different levels of magnitude; it did not only include varying levels of fairness as an important variable but also levels of authority and three alternatives for acting in the DG. Our modifications may be one reason why other studies could not find any differences in punishing behavior in individuals with schizophrenia, even though affective, cognitive, and social deficits are more pronounced in this population than in individuals on a subclinical level. Besides, inconsistent findings in previous research of altruism in schizophrenia may also result from the heterogeneous methodology of paradigms (Robson et al., 2020).

Nevertheless, there are some limitations in our study. First, although the main sample was very large (*N* = 968), only 66 individuals were assigned to the PLEs-high group. Even though most findings from the main analysis could be replicated in the matched sample (*n* = 128), small mediation effects disappeared. Second, although the idea of schizotypy as a latent personality factor with an increased risk for schizophrenia is generally accepted, one should keep in mind that only a small percentage of individuals experiencing PLEs develop schizophrenia (for meta-analyses of prevalence, see Linscott and van Os, 2013; Moreno-Küstner et al., 2018). Therefore, the present results cannot be extrapolated one-to-one to a clinical population with schizophrenia. Further, we did not control for other psychiatric conditions, which might have influenced decision-making processes. Still, the current findings provide a notable contribution in the investigation of prosocial decision-making behavior in individuals experiencing PLEs, suggesting that in addition to fairness and empathy, levels of authority, and Machiavellian personality traits may play a central role. Third, as for all experimental studies, the transfer of results to everyday life is debatable. Even though previous studies using the DG have found evidence of aberrant prosocial behavior in many psychiatric disorders (Robson et al., 2020), replication attempts are inconsistent. While some studies could not find evidence for altruistic punishment or giving in the field (Bardsley, 2008; Winking and Mizer, 2013; Balafoutas et al., 2016), others did (Balafoutas and Nikiforakis, 2012; Franzen and Pointner, 2013; Berger and Hevenstone, 2016). Further, this newly developed online experiment was presented in a somewhat abstract setting and may not generalize to real-world settings. Therefore, one should be cautious when interpreting the current results as they may differ from real social interactions. Finally, although the quality of online-studies conducted with CloudResearch has already been confirmed (McCredie and Morey, 2019), data could have been distorted by some biases like self-selection, social desirability, or demand characteristics. Nonetheless, these biases may also occur in other experimental designs conducted in other laboratories and various quality checks, which have been carried out should have reduced the risk to a minimum in the present study. Hence, future investigations of mechanisms underlying the decision-making process in economic games are needed and should consider levels of authority and Machiavellian personality traits in modified study designs.

## 5 Conclusion

To our knowledge, this was the first study investigating the role of empathy and Machiavellism in individuals experiencing high levels of PLEs in a Third-Party DG. In sum, the present study indicates that individuals experiencing high levels of PLEs do not act less prosocial than controls in general but show a somewhat aberrant decision-making behavior; particularly a pattern of intervening in *fair* situations, preferring punishment over compensation, and showing less differentiation in their behavior across contexts. Our findings suggest that people experiencing high levels of PLEs may be less able to adapt their behavior to the current situation than individuals experiencing low levels of PLEs, possibly due to deficits in social cognition. Thinking further, an unusual and harsh social interaction style (i.e., preference of punishment) could evoke interpersonal conflicts, thus compromising social functioning and possibly fueling positive symptoms such as distrust or increased tension.

Because of the vast number of methodological differences and inconsistent findings in previous research, further investigations of prosocial behavior in individuals with PLEs are crucial to better understand the underlying mechanisms of social functioning in people on the schizotypy continuum. The advantages of schizotypy research compared to investigations with clinic populations are substantial. While the cognitive and overall functioning of individuals with a chronic course of schizophrenia is often influenced by defeatist performance beliefs, stress, anxiety, physical inactivity, hospitalization, and medication effects (Moritz et al., 2021), these influences are less frequent to non-existent in individuals with subclinical manifestations of schizotypy (i.e., individuals experiencing high levels of PLEs). Therefore, schizotypy research benefits from higher generalizability in this population.

Despite various explained constraints, this work expands our understanding of prosocial behavior in individuals experiencing PLEs. It has, moreover, shown that future studies should more than before consider other variables than diagnostic status like Machiavellianism as an influencing personality trait and address the differences between empathy-guided altruistic and strategic prosocial behavior when studying social functioning in people with high-level PLEs and those at at-risk states for psychosis.

## Data availability statement

The raw data supporting the conclusions of this article will be made available by the authors, without undue reservation.

## Ethics statement

The studies involving humans were approved by The Ethics and Safety Committee of the University Medical Center Hamburg-Eppendorf (Germany). The studies were conducted in accordance with the local legislation and institutional requirements. The participants provided their written informed consent to participate in this study.

## Author contributions

JE: Conceptualization, Data curation, Formal analysis, Methodology, Project administration, Visualization, Writing – original draft, Writing – review & editing. TG: Writing – review & editing. BD: Writing – review & editing. JS: Data curation, Writing – review & editing. SM: Conceptualization, Formal analysis, Methodology, Supervision, Writing – original draft, Writing – review & editing.
