# Supplementary material for: Are psychotic-like experiences associated with aberrant prosocial decision-making behavior?
Source: Front Psychol. 2024 Aug 2;15:1387678. doi: 10.3389/fpsyg.2024.1387678 (PMC11327126; doi:10.3389/fpsyg.2024.1387678)
Supplement: Supplementary file 1 [file Data_Sheet_1.docx]

**Supplement Table S1**

*Internal consistency measurements for all questionnaire scales in both samples*

| Scale | CAPE positive | CAPE depression | TEQ | NEO-PI-R Altruism | Trimmed MACH-V | Distrust | PANAS negative | PANAS  positive |
| --- | --- | --- | --- | --- | --- | --- | --- | --- |
| Number of items | 20 | 8 | 16 | 8 | 5 | 3 | 5 | 5 |
| Cronbach’s α (N = 968) | .92 | .88 | .89 | .84 | .76 | .81 | .82 | .84 |
| Cronbach’s α (N = 128) | .94 | .84 | .88 | .80 | .73 | .71 | .82 | .78 |

**Supplement Table S2**

*Correlations of questionnaire measurements and decisions made in the DG (main sample; N = 968)*

|  | 1. | 2. | 3. | 4. | 5. | 6. | 7. | 8. | 9. | 10. | 11. | 12. | 13. | 14. | 15. | 16. | 17. |
| --- | --- | --- | --- | --- | --- | --- | --- | --- | --- | --- | --- | --- | --- | --- | --- | --- | --- |
| 1. CAPE positive |  |  |  |  |  |  |  |  |  |  |  |  |  |  |  |  |  |
| 1. CAPE depression | ,507^**^ |  |  |  |  |  |  |  |  |  |  |  |  |  |  |  |  |
| 1. Distrust | ,191^**^ | ,253^**^ |  |  |  |  |  |  |  |  |  |  |  |  |  |  |  |
| 1. MACH | ,395^**^ | ,331^**^ | ,573^**^ |  |  |  |  |  |  |  |  |  |  |  |  |  |  |
| 1. Altruism | –,132^**^ | –,198^**^ | –,182^**^ | –,289^**^ |  |  |  |  |  |  |  |  |  |  |  |  |  |
| 1. Empathy | –,262^**^ | –,156^**^ | –,180^**^ | –,375^**^ | ,636^**^ |  |  |  |  |  |  |  |  |  |  |  |  |
| 1. PANAS positive | –,010 | –,375^**^ | –,179^**^ | –,169^**^ | ,365^**^ | ,286^**^ |  |  |  |  |  |  |  |  |  |  |  |
| 1. PANAS negative | ,533^**^ | ,717^**^ | ,196^**^ | ,274^**^ | –,206^**^ | –,184^**^ | –,240^**^ |  |  |  |  |  |  |  |  |  |  |
| 1. Punish/fair | ,243^**^ | ,081^*^ | –,018 | ,057 | –,033 | –,152^**^ | ,032 | ,169^**^ |  |  |  |  |  |  |  |  |  |
| 1. Punish/unfair | ,092^**^ | ,000 | ,045 | ,066^*^ | ,013 | –,051 | ,028 | ,003 | ,117^**^ |  |  |  |  |  |  |  |  |
| 1. Punish/extreme unfair | ,052 | ,023 | ,077^*^ | ,116^**^ | ,000 | –,068^*^ | –,004 | –,006 | –,018 | ,506^**^ |  |  |  |  |  |  |  |
| 1. Compensation/ fair | ,165^**^ | ,057 | –,016 | ,058 | –,013 | –,124^**^ | –,010 | ,073^*^ | ,274^**^ | –,001 | –,068^*^ |  |  |  |  |  |  |
| 1. Compensation/ unfair | –,071^*^ | ,002 | –,052 | –,136^**^ | ,012 | ,119^**^ | ,012 | ,042 | –,024 | –,368^**^ | –,288^**^ | ,095^**^ |  |  |  |  |  |
| 1. Compensation/ extreme unfair | –,100^**^ | ,012 | –,041 | –,145^**^ | ,039 | ,164^**^ | –,017 | ,041 | –,021 | –,361^**^ | –,738^**^ | –,004 | ,496^**^ |  |  |  |  |
| 1. Keep/fair | –,238^**^ | –,080^*^ | ,021 | –,071^*^ | ,025 | ,165^**^ | –,007 | –,132^**^ | –,661^**^ | –,052 | ,061 | –,903^**^ | –,063^*^ | ,013 |  |  |  |
| 1. Keep/unfair | ,010 | –,002 | ,023 | ,095^**^ | –,022 | –,087^**^ | –,032 | –,045 | –,055 | –,301^**^ | –,048 | –,097^**^ | –,776^**^ | –,264^**^ | ,100^**^ |  |  |
| 1. Keep/extreme unfair | ,076^*^ | –,047 | –,042 | ,058 | –,056 | –,146^**^ | ,029 | –,051 | ,053 | –,142^**^ | –,244^**^ | ,095^**^ | –,338^**^ | –,475^**^ | –,098^**^ | ,442^**^ |  |

Note.

** p < .01 (2-sided). * p < .05 (2-sided).

**Supplement Figure S3**

*Mediation analyses for compensation and punishment rates in fair, unfair and extremely unfair trials (matched sample; N = 128)*

*Note*. *Note*. Models of mediation analyses (secondary analysis) with schizotypy as predictor, empathy and Machiavellism as mediators, and (a) compensation and (b) punishment rates in different fairness conditions as criterions. Beta-coefficients and standard errors for direct effects are displayed next to regression pathways.

* *p* < .05. ** *p* < .01. *** *p* < .005. **** *p* < .001.“
